# Supplementary material for: An investigation of plasma interleukin-6 in sport-related concussion
Source: PLoS One. 2020 Apr 28;15(4):e0232053. doi: 10.1371/journal.pone.0232053 (PMC7188239; doi:10.1371/journal.pone.0232053)
Supplement: Attachment — Submitted filename: Response to Reviewers PLOS.docx [file pone.0232053.s001.docx]

**Response to Reviewers: Answers highlighted in green.**

When submitting your revision, we need you to address these additional requirements:

1. Please ensure that your manuscript meets PLOS ONE's style requirements, including those for file naming. The PLOS ONE style templates can be found at <http://www.plosone.org/attachments/PLOSOne_formatting_sample_main_body.pdf> and<http://www.plosone.org/attachments/PLOSOne_formatting_sample_title_authors_affiliations.pdf>

2. We note that you have indicated that data from this study are available upon request. PLOS only allows data to be available upon request if there are legal or ethical restrictions on sharing data publicly. For information on unacceptable data access restrictions, please see <http://journals.plos.org/plosone/s/data-availability#loc-unacceptable-data-access-restrictions>.

Response: We have now uploaded our data for readers.

In your revised cover letter, please address the following prompts:

a) If there are ethical or legal restrictions on sharing a de-identified data set, please explain them in detail (e.g., data contain potentially identifying or sensitive patient information) and who has imposed them (e.g., an ethics committee). Please also provide contact information for a data access committee, ethics committee, or other institutional body to which data requests may be sent.

Response: There are none, we have now uploaded our dataset

b) If there are no restrictions, please upload the minimal anonymized data set necessary to replicate your study findings as either Supporting Information files or to a stable, public repository and provide us with the relevant URLs, DOIs, or accession numbers. Please see <http://www.bmj.com/content/340/bmj.c181.long> for guidelines on how to de-identify and prepare clinical data for publication. For a list of acceptable repositories, please see <http://journals.plos.org/plosone/s/data-availability#loc-recommended-repositories>.

 Response: We have uploaded our dataset to the Harvard dataverse.

Link: <https://doi.org/10.7910/DVN/THZCRP>

Please advise if there are any formatting issues.

We will update your Data Availability statement on your behalf to reflect the information you provide.


Reviewer's Responses to Questions

**Comments to the Author**

1. Is the manuscript technically sound, and do the data support the conclusions?

The manuscript must describe a technically sound piece of scientific research with data that supports the conclusions. Experiments must have been conducted rigorously, with appropriate controls, replication, and sample sizes. The conclusions must be drawn appropriately based on the data presented. 

Reviewer #1: Yes

2. Has the statistical analysis been performed appropriately and rigorously? 

Reviewer #1: I Don't Know

3. Have the authors made all data underlying the findings in their manuscript fully available?

The [PLOS Data policy](http://www.plosone.org/static/policies.action#sharing) requires authors to make all data underlying the findings described in their manuscript fully available without restriction, with rare exception (please refer to the Data Availability Statement in the manuscript PDF file). The data should be provided as part of the manuscript or its supporting information, or deposited to a public repository. For example, in addition to summary statistics, the data points behind means, medians and variance measures should be available. If there are restrictions on publicly sharing data—e.g. participant privacy or use of data from a third party—those must be specified.

Reviewer #1: Yes

4. Is the manuscript presented in an intelligible fashion and written in standard English?

PLOS ONE does not copyedit accepted manuscripts, so the language in submitted articles must be clear, correct, and unambiguous. Any typographical or grammatical errors should be corrected at revision, so please note any specific errors here.

Reviewer #1: Yes

5. Review Comments to the Author

Please use the space provided to explain your answers to the questions above. You may also include additional comments for the author, including concerns about dual publication, research ethics, or publication ethics. (Please upload your review as an attachment if it exceeds 20,000 characters)

Reviewer #1: This is an interesting study and the motivation of this work is clearly explained. The paper is generally well written. However, in my opinion, the paper is redundant, in particular the results of two papers [ref. 4,5] are repeated several times in the introduction (page 4, line 20-23; page 5, lines 21-23; page 6 lines 1-2) and discussion paragraphs. I therefore recommend restructuring them, making a mention of the previous citations (“as previously mentioned”) and to cite them fewer times in order to avoid redundancy.

Response: Thank you for bringing this to our attention. The reason for the repeated citing of the studies by Merchant Borna and Nitta, respectively, are that they are the only two papers in the sport-concussion space which have looked at IL-6 following injury and throughout recovery. Hence, we felt it important and unavoidable to cite these papers throughout the introduction and discussion, as they required a more in-depth appraisal and comparison. However, we agree that there is an appearance of redundancy when the manuscript is read as a whole. While we continue to cite these papers when mentioned in-text (especially when new information is introduced in the sentence) we have adjusted the introduction (page 5 lines 22-23, page 6 line 1) and discussion (page 15, lines 9-14 & 18-20) by reducing author names in-text, removing redundant information pertaining these references where possible, and including the statements “as previously mentioned” or “aforementioned study”.

Below I have provided some minor remarks on the text.
Please, control all the abbreviations. For example: page 2, line 6, IL-6 abbreviation should be identified at the first appearance in the text; pag 4, line 19, the abbreviation of SRC has already been introduced in pag 2, line 7; pag 5, lines 2-3, the abbreviation of mTBI has already been introduced in pag 4, line 3, pag 5, line 20, spell-out CSF.

Response: Thank you for bringing this to our attention. We have revised each of the above abbreviation errors (mTBI: pg 5, line 3; IL-6: pg 2 line 6, pg 2 line 13, pg 5 line 13) and have spelled out CSF (pg 5 line 20). However, we left the complete identification of SRC on page 4 line 18, as this is the first manuscript appearance (pg 2 line 7 is the abstract, we traditionally treat this as a stand-alone section similar to a figure legend).

Pag 4, lines 20-23. It may be relevant to specify which genes are up-regulated and which down-regulated compared with control (specify which control).

Response: Please see updated information added to up page 4, line 21, that includes a list of the most important genes modulated following injury: all genes could not be stated, as there were many that were altered. We also added in the control comparison for these differences on page 4 lines 22-23.

Pag 6, lines 2-4. Please clarify sentence. What do the authors mean by "as opposed to circulating protein concentrations "?

Response: This was meant to articulate the difference between the findings of Merchant Borna et al., who looked at the mRNA inside immune cells vs. the actual circulating concentrations of inflammatory proteins (cytokines, chemokines etc.,).

Pag 7, lines 1-5. The total number of participants for individual sport is 97, not 96.

Response: We apologize for this error. There are indeed 96 participants in the study. The additional male hockey player was erroneously in Table 1 and in text as a legacy issue: when we began processing the data there were 97 participants, but one was mistakenly labelled as a concussion (male hockey) when in fact this person had an eye injury. This has now been corrected in both Table 1 and on page 7, lines 1-5.

Pag 8, line 2. “Blood” instead “plasma”.

Response: Thank you for pointing this out, this now reads plasma on page 8, line 2.

Pag 12, Table 1. Again, the total number of SRC for individual sport is 42, not 41. Please, recalculate the percentages or clarify why a recruited subject has been removed from the total. If necessary, also correct the total number of participants and/or SRC individuals in the abstract.

Response: Please see explanation above, this has now been corrected.

Please, add a point at the end of the Table 1 title and uniform the descriptive legend with the other two tables, using first the abbreviation and then the spelled-out form.

Response: Please see revisions to Table 1 title and legend.

Pag 15, line 18. Add dash in IL6.

Response: Please see added dash on page 15, line 18.

6. PLOS authors have the option to publish the peer review history of their article ([what does this mean?](https://journals.plos.org/plosone/s/editorial-and-peer-review-process#loc-peer-review-history)). If published, this will include your full peer review and any attached files.


If you choose “no”, your identity will remain anonymous but your review may still be made public.


**Do you want your identity to be public for this peer review?** For information about this choice, including consent withdrawal, please see our [Privacy Policy](https://www.plos.org/privacy-policy).

Reviewer #1: No
